# Association of subjective memory complaints amid patients of Diabetes Mellitus Type II and Hypertension

**DOI:** 10.12669/pjms.37.2.3426

**Published:** 2021

**Authors:** Samira Faiz, Farhan Muhammad Qureshi, Amreen Wasif Hussain, Seema Nigah-e-Mumtaz

**Affiliations:** 1 Dr. Samira Faiz, MPH. Senior Lecturer, Department of Community Health Sciences, Karachi Institute of Medical Sciences, Malir Cantt, Karachi, Pakistan; 2 Dr. Farhan Muhammad Qureshi, MS - Public Health & Health Promotion Assistant Professor, Department of Community Health Sciences, Karachi Institute of Medical Sciences, Malir Cantt, Karachi, Pakistan; 3 Amreen Wasif Hussain, MPH. Programme Assistant, Global Health Directorate, Indus Health Network, Karachi, Pakistan; 4 Prof. Dr. Seema Nigah-e-Mumtaz, MPH, DCPS-HCSM. Department of Community Health Sciences, Karachi Institute of Medical Sciences, Malir Cantt, Karachi, Pakistan

**Keywords:** Dementia, Diabetes, Hypertension, Subjective memory complaints

## Abstract

**Background and Objective:**

Subjective memory complaints (SMCs) are suggested to predict dementia at a very early stage. However, association of SMCs with known risk factors of dementia namely diabetes mellitus Type-2 diabetes and hypertension (HTN) remain unexplored which is the main aim of this study. The objective of the study was to investigate the association of SMC with diabetes mellitus type 2 (DM2) and hypertension (HTN).

**Methods:**

The associations of diabetes and hypertension, with SMCs has been tested in 500 participants from a tertiary care hospital of Karachi during 2017. Diagnosed cases of diabetes and hypertension were included through convenient sampling. Healthy attendants of patients were interviewed for the reference group. SMCs were assessed through a 14-item SMC questionnaire.

**Results:**

Sample included 114 patients with only diabetes and hypertension each, 103 with both diabetes and hypertension and 169 healthy participants. Compared to healthy adults, persons with diabetes and hypertension had higher SMCs (difference, 0.88, 95% CI: 0.22, 1.54) (difference, 1.06, 95% CI: 0.40, 1.71) respectively, in fully adjusted models.

**Conclusions:**

Compared to healthy adults of working age-group, persons with diabetes and hypertension were more likely to have SMCs. Assessment and early detection of SMCs in persons with diabetes and hypertension might be informative to provide a window for effective interventions to maintain cognitive health.

## INTRODUCTION

In the present era, there is an anticipated increase in dementia worldwide, for which the major contributor is the high prevalence of diabetes and hypertension which are known risk factors of brain pathology.^1^,^2^ Since, no curative treatment for dementia is available to date, the focus remains on detecting the disease in its preclinical phase, so that meaningful interventions could slow their progression. Currently, subjective memory complaints (SMCs) have been of considerable interest to researchers and clinicians for their role in predicting various neurological conditions such as stroke and dementia.^3^ A common underlying pathology for these disorders is vascular pathology and cardiovascular risk factors that ultimately result in cognitive impairment and dementia.^4^,^5^ As dementia follows a long preclinical phase, SMCs might be an early indicator on the continuum of cognitive decline. A further step could be to explore the association of SMCs with highly prevalent known risk factors of dementia, i.e. diabetes and hypertension which is the main aim of this study. Therefore, our core objective to conduct this research was to investigated the association of diabetes and hypertension with SMCs in ambulatory participants visiting outpatient medical clinics in a tertiary care hospital setting which would thus detect cognitive damage even in an earlier stage of diabetes.

## METHODS

The study was conducted in a tertiary care hospital of Karachi in 2017. Data were collected from 500 participants through convenient sampling after getting approval from Ethical Review Board of Karachi Institute of Medical Sciences (Ref No. ERC-KIMS/002/17, dated June 10, 2017). After an informed verbal consent, only diagnosed patients with diabetes and/or hypertension (since not less than a year) were further investigated for other comorbidities through medical records maintained by the hospital; while information about pregnancy/puerperium, and/or history of severe head injury, diagnosed dementia or stroke was collected through self-report. Those found positive for these conditions were excluded. For the recruitment of the reference/control group healthy attendants of patients were interviewed who did not have any history of medical and surgical illness and/or medications on self-report. For further confirmation, random blood sugar levels were measured using Glucometer and blood pressure was recorded. At this stage, 507 participants were enrolled.

Finally, written informed consent was then obtained by all eligible participants. SMCs were assessed by the Subjective Memory Complaints Questionnaire (SMCQ) while objective memory impairment was assessed by Mini-Mental State Examination (MMSE)^6^ to rule out any Mild Cognitive Impairment (MCI) and early stages of dementia. Seven participants scored high (≥5/14) on SMCQ and low (≤24/30) on MMSE were excluded from the study. This resulted in a dementia- and MCI-free sample of 500 participants. Subsequently, in a detailed interview, information on socio-demographic characteristics, anxiety (GAD-7) and depression (PHQ-9) was collected. Height and weight were recorder after the interview.

### Subjective memory complaints questionnaire (SMCQ)

SMCQ was developed by JC Youn consisting of 14 items, with a minimum score of 0 (absence of complaint) and maximum score of 14. First four items assess global memory function while the latter ten assess working memory function. Higher scores indicate worse memory. In addition, the cut-off of five can be used to dichotomize participants for having or not having SMCs for descriptive purposes.^7^,^8^

### Statistical analyses

Comparisons were made using t-tests and Analysis of Variance (ANOVA) for continuous variables and chi-square test for categorical variables. All tests were two-tailed, and level of significance was set at 0.05. Association with SMCs as continuous outcome was done using multiple linear regression while categorized SMC score was analyzed using logistic regression models. Analyses were performed using IBM SPSS statistics version 21.0 (IBM Corp., Armonk, N.Y., USA).

## RESULTS

Out of 500 participants, 114 cases of diabetes and hypertension each, 103 cases having both diabetes and hypertension while 169 healthy adults. The mean age was 48±9.2 years and 47.2% were women. Majority of the study population was married (85%). 15% of the study population was unable to read or write, while 16% had postgraduate education. Almost half of the population was working, and mostly held a desk job. 86% never smoked while the mean BMI (kg/m²) was 26.9±5.2. Approximately, 10% of the participants were found to be having anxiety, depression or both (Table-I).

**Table-I:** Comparison of characteristics between the reference and diseased groups, N=500.

*Characteristics*		*Descriptive*			

*Socio-Demographics*		*Reference group n=169*	*Diabetic group n=114*	*Hypertensive group n=114*	*Hypertensive & diabetic group n=103*
Age	Years mean (SD)	43.5 (8.6)	49.4 (8.6)	48.6 (8.8)	53.2 (8.0)
Gender	Women n (%)	62 (36.7)	46 (40.3)	70 (29.7)*	58 (24.6)*
Married	Yes n (%)	139 (82.2)	102 (89.5)	100 (87.7)	84 (81.6)
Education n (%)	Not able to read/write	9 (5.3)	23 (20.2)*	15 (13.2)*	30 (29.1)*
	Primary, < 5 years	16 (9.5)	15 (13.1)*	15 (13.2)*	10 (9.7)
	Secondary, 5-12 years	47 (27.8)	40 (35.1)*	35 (30.7)*	30 (29.1)*
	Graduate, up to 16 years	54 (31.9)	25 (21.9)*	33 (28.9)	22 (21.4)*
	Postgraduate, >16 years	43 (25.4)	11 (9.6)*	16 (14.0)*	11 (10.7)*
Employment	Yes vs No n (%)	117 (69.2)	58 (50.9)*	43 (37.7)*	34 (33.0)*
Occupation n (%)	Not working/ Retired	42 (24.8)	47 (41.2)	67 (58.8)	66 (64.1)
	Desk work	66 (39.0)	34 (29.8)	23 (20.2)	21 (20.4)
	Skilled work	37 (21.9)	24 (21.0)*	32 (28.1)*	11 (10.7)
	Manual work	17 (10.1)	7 (6.1)	11 (9.6)*	4 (3.9)*
	Others	4 (2.4)	2 (1.7)	1 (0.9)	1 (1.0)*
Smoker	Yes n (%)	16 (9.5)	11 (9.6)	9 (7.9)	8 (7.8)
BMI†(kg/m²)	mean (SD)	25.3 (4.6)	27.6 (5.0)*	27.0 (5.6)*	28.5 (5.5)*

* Indicates that a group differs significantly (p-value <0.05) from the reference.

† Body Mass Index.

The association of socio-demographic factors with SMCs based on linear regression models and mutually adjusted for all variables are presented in Table-II. Adjusting for each depression and anxiety in separate models along with all other covariates had similar results, therefore, only results adjusted for depression along with all covariates are presented.

**Table-II:** Association b/w socio-demographic factors & subjective memory complaints N=500.

*Socio-Demographics*		*Subjective Memory Complaints*		

*Variables*		*Difference (β)*	*95% CI*	*P-value*
Age	Years	0.05	0.01, 0.08	0.008
Gender	Women	0.66	-0.15, 1.48	0.11
Married	Yes	1.40	-0.14, 2.93	0.07
Education	Not able to read/write	Reference		
	Primary, < 5 years	-0.36	-1.52, 0.80	0.54
	Secondary, 5-12 years	-1.25	-2.21, -0.29	0.01
	Graduate, up to 16 years	-1.28	-2.30, -0.27	0.01
	Postgraduate, >16 years	-1.81	-2.99, -0.62	0.003
Employment	Yes vs. No	-1.78	-2.89, -0.67	0.002
Occupation	Not working/Retired	Reference	
	Desk work	0.76	-0.50, 2.01	0.23
	Manual work	1.97	0.56, 3.38	0.006
BMI †	Kg/m^2^	0.08	0.02, 0.14	0.007

† Body Mass Index

The association of diabetes, hypertension, and comorbid diabetes and hypertension with SMCs are presented in Table-III. After additionally adjusting for depressive (model 3) and anxiety (model 4) symptoms, results attenuated but remained significant. We did not adjust for both depression and anxiety in the same model due to their high correlation (r=0.92). Using SMC as a categorical variable with a cut-off score of 5 and observed consistent results (Table-IV).

**Table-III:** Association of diabetes mellitus type 2 (DMT2) and hypertension (HTN) with subjective memory complaints score N=500.

*Subjective Memory Complaints*								

*Variable*	*Model 1*		*Model 2*		*Model 3*		*Model 4*	

	*Difference (95% CI)*	*P-value*	*Difference (95% CI)*	*P-value*	*Difference (95% CI)*	*P-value*	*Difference (95% CI)*	*P-value*
DMT2 Yes vs. No	1.50(0.87, 2.14)	<0.001	1.10 (0.44, 1.75)	0.001	0.88 (0.22, 1.54)	0.009	0.84 (0.18, 1.50)	0.01
HTN Yes vs. No	1.45 (0.80, 2.09)	<0.001	1.21 (0.56, 1.86)	<0.001	1.06 (0.40, 1.71)	0.002	1.11 (0.46, 1.75)	0.001
DMT2 + HTN Yes vs. No	2.65 (1.72, 3.59)	<0.001	1.96 (0.92, 3.00)	<0.001	1.63 (0.57, 2.69)	0.003	1.64 (0.60, 2.68)	0.002

Model 1: adjusted for age and sex. Model 2: Model 1 + body mass index, education, marital status, working status, occupation, and smoking.

**Table-IV:** Association of diabetes mellitus Type 2 (DMT2) and hypertension (HTN) with presence of subjective memory complaints N=500.

*Variable*	*Model 1*		*Model 2*	

	*Odds ratio (95% CI)*	*P-value*	*Odds ratio (95% CI)*	*P-value*
DMT2 Yes vs. No	2.34 (1.58, 3.45)	<0.001	2.14 (1.40, 3.30)	0.001
HTN Yes vs. No	2.15 (1.46, 3.17)	<0.001	2.00 (1.31, 3.06)	0.001
DMT2 + HTN Yes vs. No	4.57 (2.45, 8.53)	<0.001	3.58 (1.71, 7.48)	0.001

Model 1: adjusted for age and sex., Model 2: additionally, adjusted for body mass index, education, marital status, working status, Occupation, & smoking. Analyses of diabetes are additionally adjusted for HTN & vice versa.

In another sensitivity analyses, the association between diabetes and SMC has been tested after excluding persons with hypertension and vice versa. Results were consistent and stronger; persons with diabetes were more than thrice as likely to have SMC (OR 3.31, 95% CI: 1.85, 5.92) and had a higher SMC score as compared to those without diabetes, (difference (β) 1.88, 95% CI: 1.03, 2.73) in fully adjusted models. Similarly, results for hypertension also became stronger; persons with hypertension were thrice as likely to have SMC (OR 3.06, 95% CI: 1.73, 5.39), and had a higher SMC score as compared to those without hypertension, (difference (β) 1.99, 95% CI: 1.14, 2.83).

## DISCUSSION

This research concludes that people with diabetes or hypertension were more likely to have SMCs than those without diabetes or hypertension. Independent of socio-demographic factors, cardiovascular risk factors, and depression and anxiety, persons with comorbid diabetes and hypertension were even more likely to have SMCs than with diabetes or hypertension alone. We found that having diabetes was associated strongly with SMCs, and hypertension, education, working status, depression and anxiety were important confounders. Age, sex, BMI and smoking which are conventional cardiovascular risk factors were not important confounders in our study. Since there are no studies that have investigated the association of diabetes and/or hypertension with SMCs, comparison of results could not be made. However, epidemiological studies have shown high co-occurrence of diabetes and dementia.^2^

Reviewing cognitive decline among patients with diabetes; research reported diabetes-associated cognitive decrements, subtle cognitive changes that occur progressively among patients with diabetes.^9^ These are not as severe as dementia however, cause serious implications on patients as they age (mostly >65 years of age).^9^ Results of this study indicates that the neurodegenerative changes start at a very early stage in persons with diabetes which reflects as SMC. These changes may lead to cognitive decline and dementia as the age progresses.

Similarly, we found that hypertensive individuals were more likely to have SMCs than their healthy counterparts. Hypertension has been related to the development of dementia, including Alzheimer’s disease, and cognitive dysfunction in middle-aged and elderly populations.^10^,^11^ Findings of this research suggest that hypertension causes neurodegenerative changes in midlife, these micro-vascular damages are associated with hypertension. The Shanghai aging study found consistent association between elevated blood pressure and late-life cognitive impairment.^12^ Blood pressure fluctuations or consistently uncontrolled hypertension over many years might be more predictive of SMC but we did not have longitudinal data.

Persons with comorbid diabetes and hypertension were even more likely to have SMCs than with any one condition alone. This additive risk results are plausible that comorbid diabetes and hypertension may poses an added risk for dementia and other cardiovascular events as well.^13^,^14^ Our results thus indicate their additive effect, which manifests itself at a very early stage in the continuum of cognitive damage. This means that persons with both diabetes and hypertension should be treated more vigorously for their diabetes and hypertension as they are possibly at a much higher risk of developing neurodegenerative changes than those with diabetes or hypertension alone. The vigorous intervention should include medication as well as overall healthy lifestyle such as physical, mental and social activity, refraining from smoking or alcohol, and a balanced diet.

Several studies suggest that education and occupational complexity are associated with lower risk of dementia.^15^,^16^ Therefore, a possible explanation could also be the cognitive reserve hypothesis, which states that persons with higher education and who are working can tolerate more insults to the brain before they become clinically apparent.^17^,^18^

Finally, depression and anxiety have been also reported to be a consequence of same pathological pathways as neurodegeneration,^19^,^20^ and therefore it has been observed as a significant predictor of SMCs. However, even after adjusting for them, diabetes and hypertension remained significantly associated with SMCs.

### Strengths and Limitations

This is perhaps the first study in Pakistan investigating the association of diabetes and hypertension with SMCs, particularly, among the working age group. Depression and anxiety, being important confounders, were also considered and adjusted for the results. Any possible case of MCI or dementia was excluded through objective memory assessment. However, relying on self-report for certain risk factors might have resulted in some information bias. Moreover, residual confounding might be possible due to of unknown confounders, such as physical activity and nutrition. However, we did adjust for body mass index as a proxy of overall physical health.

## CONCLUSION

Working age-group persons with diabetes and hypertension are more likely to have SMCs than their healthy counterparts. Persons having comorbid diabetes and hypertension are even more likely to have SMCs. Assessment of SMCs in persons with diabetes and hypertension might be informative and provide a window for effective interventions to maintain cognitive health.

### Author’s Contribution:

**SF:** Concept & design of project, literature review, statistical analysis, manuscript write up, take the overall responsibility & accountable for the accuracy or integrity of the work.

**FMQ & AWH:** Data collection, statistical analysis and interpretation of results, critically reviewed and edited the manuscript

**SNM:** Research Supervisor and final approval of the manuscript.
